# Correction: Pharmacophore modeling, docking and the integrated use of a ligand- and structure-based virtual screening approach for novel DNA gyrase inhibitors: synthetic and biological evaluation studies

**DOI:** 10.1039/d6ra90082h

**Published:** 2026-07-29

**Authors:** Deepti Mathpal, Mukesh Masand, Anisha Thomas, Irfan Ahmad, Mohd Saeed, Gaffar Sarwar Zaman, Mehnaz Kamal, Talha Jawaid, Pramod K. Sharma, Madan M. Gupta, Santosh Kumar, Swayam Prakash Srivastava, Vishal M. Balaramnavar

**Affiliations:** a Sanskriti University, School of Pharmacy and Research 28 KM. Stone, Mathura – Delhi Highway, Chhata Mathura Uttar Pradesh (UP) 281401 India v.balaramnavar@gmail.com; b Department of Pharmacy, Faculty of Medicine and Allied Sciences, Galgotias University Gautam Buddha Nagar Uttar Pradesh 226001 India; c Department of Chemistry, School of Advanced Sciences, VIT Vellore India; d Department of Clinical Laboratory Science, College of Applied Medical Sciences, King Khalid University Abha Saudi Arabia; e Department of Biology College of Sciences, University of Hail Saudi Arabia; f Department of Pharmaceutical Chemistry, College of Pharmacy, Prince Sattam Bin Abdulaziz University P.O. Box No. 173 Al Kharj 11942 Kingdom of Saudi Arabia; g Department of Pharmacology, College of Medicine, Al Imam Mohammad Ibn Saud Islamic University (IMSIU) Riyadh 13317 Kingdom of Saudi Arabia; h School of Pharmacy, Faculty of the West Indies St Augustine West Indies; i Government Degree College Hansaur Barabanki Uttar Pradesh (UP) 225415 India; j Department of Pediatrics, Yale University School of Medicine New Haven CT 06520 USA; k Vascular Biology and Therapeutic Program, Yale University School of Medicine New Haven CT 06511 USA

## Abstract

Correction for ‘Pharmacophore modeling, docking and the integrated use of a ligand- and structure-based virtual screening approach for novel DNA gyrase inhibitors: synthetic and biological evaluation studies’ by Deepti Mathpal *et al.*, *RSC Adv.*, 2021, **11**, 34462–34478. https://doi.org/10.1039/D1RA05630A.

The authors regret that the author contributions statement did not accurately reflect the contributions of the authors. The correct Author contributions statement is shown below:

**Author contributions**

Dr Deepti Mathpal: Dr Deepti had a role in the study’s conceptualization. She collected compounds from the literature, prepared the defined database, drew their structures and prepared ligands ready for further study. She also participated in writing the first draft of the manuscript.

Dr Mukesh Masand: Dr Mukesh had a role in the study’s conceptualization. He was involved in the synthesis and of characterization of compounds.

Dr Anisha Thomas: Dr Anisha had a role in the study’s conceptualization. She was responsible for pharmacophore modelling, scaffold hopping, docking studies and ligand prioritization.

Dr Irfan Ahmad: Dr Irfan secured the funding for this project and arranged resources used in this work. Dr Irfan assisted with the *in silico* studies, provided access to the software, analyzed the results, and gave feedback on the manuscript.

Dr Mohd Saeed: Dr Saeed had a role in data curation. He provided software (Biovia previously known as Discovery Studio 3.1) support and interpreted the output (biological and computational) data.

Dr Gaffar Sarwar Zaman: Dr Zaman had a role in the data from docking analysis and interpretation.

Dr Mehnaz Kamal: Dr Mehnaz investigated and visualized the results of the docking experiments.

Dr Talha Jawaid: Dr Talha assisted in the chemical synthesis of the reported compounds. He also had a role in the visualization and interpretation of the data on the compounds.

Dr Pramod K. Sharma: Dr Pramod assisted in providing the resources for the chemical synthesis of the reported compounds.

Dr Madan M. Gupta: Dr Madan assisted in optimization of the synthesis and product purification.

Dr Santosh Kumar: Dr Santosh assisted in the synthetic methodology optimization. He also supervised the write up of the synthetic part of this work.

Dr Swayam Prakash Srivastava: Dr Swayam had a role in the biological evaluation and supervision of the project.

Dr Vishal M. Balaramnavar: Dr Vishal had a role in the study’s conceptualization. He performed the pharmacophore development, result validation, and mapping studies. He also had a role in the project administration.

The affiliation of the corresponding author, Sanskriti University, has verified the contributions and confirmed that this accurately reflects the contributions of the authors.

The Royal Society of Chemistry apologises for these errors and any consequent inconvenience to authors and readers.

